# The Association Between Teenage Pregnancy and the Development of Asthma in Children Aged Zero to Five Years

**DOI:** 10.7759/cureus.88158

**Published:** 2025-07-17

**Authors:** Alejandra Viera Plasencia, Lisett Castellanos, Valentina Roa Forster, Juan Ruiz-Pelaez

**Affiliations:** 1 Division of Medical and Population Health Sciences Education and Research, Florida International University, Herbert Wertheim College of Medicine, Miami, USA; 2 Department of Research, Kangaroo Foundation, Bogota, COL

**Keywords:** childhood asthma, maternal age, pediatric health outcomes, social determinants of health (sdoh), teenage pregnancy

## Abstract

Introduction and objective

Childhood asthma is a significant public health concern and the most common chronic disease in children, particularly affecting those under five. Maternal factors, including age at delivery, may influence asthma risk, as teenage mothers often face socioeconomic challenges that impact child health. This study examines the association between maternal age at delivery (teenage vs. adult mothers) and childhood asthma.

Methods

This historical cohort study used secondary data from the 2021 National Survey of Children’s Health. Children aged zero to five were included, excluding those born to mothers over 45, children with congenital defects, and those with a maternal asthma history. Multiple logistic regression was used to estimate adjusted odds ratios (OR, 95% CI) for asthma.

Results

Among 48,379 children, 1,110 (2.29%) were born to teenage mothers (≤19 years). Asthma prevalence was higher in children of teenage mothers (9.01%) than adult mothers (4.28%) (p < 0.001). Before adjustment, children of teenage mothers had more than twice the odds of having asthma compared to those of adult mothers (OR 2.21, 95% CI 1.79-2.73). After adjustment, the odds remained significantly higher (OR 1.39, 95% CI 1.09-1.77).

Conclusion

Children of teenage mothers have increased odds of developing asthma. Future research should explore mediating factors, such as socioeconomic and environmental determinants, to clarify this association. These findings highlight the need for targeted healthcare policies, such as improved access to prenatal care, mental health support, and parental education programs, to support teenage mothers and mitigate early childhood health risks.

## Introduction

Childhood asthma is considered a serious public health issue globally, and it is thought to be the most common chronic disease among children [1]. According to the World Health Organization (WHO), death rates from asthma in the pediatric population range from zero to 0.7 per 100,000 people, and the asthma prevalence is expected to reach 400 million cases by 2025 [2]. Even though the mortality rate for this disease is low, the morbidity remains elevated [2]. According to the Centers for Disease Control and Prevention (CDC), one in 12 children between the ages of zero and 17 has asthma, and the attacks are more frequent in children younger than age five. Additionally, emergency department and urgent care center visits due to asthma attacks are more common in children between zero and four years old [3].

According to the literature, asthma is a multifactorial disease, and factors such as age, sex, family history, environment, and behaviors are disease triggers [4]. Asthma risk begins during fetal development and continues through early childhood, particularly during the first two years of life, as the child’s immune system begins to mature [4]. Additionally, many of the factors that contribute to the development of asthma in childhood are also associated with its persistence into adulthood [5]. Teenage mothers frequently face chronic stress due to socioeconomic instability, limited healthcare access, and psychosocial challenges. Maternal stress during pregnancy leads to increased cortisol exposure in the fetus, which may be associated with the promotion of the Th2 immune response at birth, leading to increased risk of childhood asthma [6].

Teenage pregnancies are one of the major global public health problems, according to the United Nations Population Fund (UNFPA). This also relates to low birth weights, low Apgar scores (birth asphyxia), and perinatal mortality [7]. The US has the highest frequency of teenage pregnancies among high-income countries [8]. According to research published by the Royal Australian College of General Practitioners, teenagers who become pregnant are more likely to be from lower socioeconomic status and have many social, medical, and financial difficulties [9]. As mentioned previously, the article also supports the high prevalence of low birth weight and postnatal comorbidities in children born to teenage mothers, as opposed to adult mothers [9].

Factors more prevalent among teenage mothers, such as high levels of anxiety or stress, can be associated with the development of asthma in children [10]. Stress leads to elevated cortisol, and abnormal levels of cortisol have been associated with wheezing in children [11]. Additionally, stress in the mother can result in epigenetic changes that affect the development of the child [12].

Asthma is a very common childhood disease, and there are many factors during pregnancy and early childhood that contribute to the development of asthma [4]. Current literature focuses on the risk factors of asthma development, such as smoking in the house, being exposed to different triggers or allergens, deficiencies, etc. However, while specific prenatal and early-life risk factors for asthma (like smoke exposure or allergens) are well-documented, limited research has focused specifically on the association between maternal age at pregnancy and the development of asthma in children. Some studies have begun to explore this link, but findings remain limited and inconsistent. Given the toll that teenage pregnancy and asthma have on the healthcare system and the associated risk factors, it is important to study their association [13,14].

Given these environmental and biological factors present in a teenage mother, as well as the multifactorial causal pathways leading to asthma, this research aims to explore the association between the incidence of asthma in children and teenage pregnancy.

## Materials and methods

Study design

The study design was a historical cohort study involving a secondary data analysis of the National Survey of Children’s Health (NSCH) database.

Data source

This study used the NSCH database. This survey from the US Department of Health and Human Services logs information regarding the health of children in the US, as well as factors that may influence children’s health, such as family, school, and neighborhood. The survey is administered randomly through email or mail to different households with children across the 50 states of the US. The survey is completed by the caregiver of the child (biological or adoptive parents, foster parents, grandparents, step-parents, and other relatives or non-relatives).

Study population

All children aged zero to five years who were born to teenage or adult mothers and recorded in the NSCH database between 2018 and 2021 from any US state were included in this study, provided they met the inclusion criteria. This age group was selected because it represents a critical developmental period when asthma often first presents, and it aligns with the predefined zero-to-five-year category used in the NSCH dataset, allowing for consistent data extraction and analysis. Data from all four survey years (2018-2021) were pooled to increase statistical power. The NSCH provides complex survey weights to ensure national representativeness; these weights were applied in all analyses to account for stratification, clustering, and nonresponse, following NSCH analytic guidelines.

Inclusion criteria

Children between zero and five years of age in the US, who were reported in the NSCH between 2018 and 2021, with valid information about asthma status. Born to mothers with ages ranging from less than 20 years (teen mothers) to 20 to 44 years of age (adult mothers), as reported in question B4.

Exclusion criteria

Children were excluded if they had congenital birth defects, as identified through caregiver responses to relevant NSCH survey items, in order to reduce confounding due to underlying medical conditions that may independently influence asthma risk. We also excluded children whose caregivers were not biological or adoptive parents to improve the accuracy of reported maternal characteristics and to maintain biologically relevant mother-child data pairs for analysis.

Variables

The exposure variable was the age of the mother at the time of birth, categorized as less than 20 years (teen pregnancy) versus 20 to 44 years (adult pregnancy). This categorization is consistent with the CDC and WHO definitions of adolescent pregnancy. The outcome was the presence or absence of asthma in children aged zero to five years, as reported by the caregiver in response to question A6 of the NSCH survey.

Control variables included the marital status of caregiver, sex of caregiver, socioeconomic status, level of education of caregiver, location of birth of the mother and age of immigration, location of birth of the child and age of immigration, social determinants of health (such as household income, neighborhood safety, and parental employment), access to healthcare or regularity of health checks, homelessness or lived in shelter, smokers in the house, child’s overall health, respiratory problems, allergies in the child, fetal alcohol spectrum disorder, infant age, feeding, health, overweight child, time spent by the child outdoors, and comorbidities.

Although the database provided extensive sociodemographic and health-related variables, it lacked direct measures of certain well-established risk factors for asthma. Variables such as maternal smoking during pregnancy, family history of asthma or atopy, and exposure to indoor allergens were not explicitly captured. While we adjusted for proxy variables including household smoking exposure, caregiver education, and socioeconomic status, the absence of these direct measures may introduce residual confounding. This limitation should be considered when interpreting the findings, as these factors could influence the observed association between maternal age and childhood asthma.

Statistical analysis

Data were obtained from the NSCH Questionnaire for children aged zero to five years. We began with an exploratory analysis to describe the distribution of variables, identify missing data, and recode variables as necessary. Cases with missing data on the exposure, outcome, or key control variables were excluded using listwise deletion. Bivariate analyses were then conducted to evaluate the distribution of control variables in relation to maternal age group (teen vs. adult mothers), as well as their association with asthma outcomes. Chi-square tests were used for categorical variables, and t-tests or non-parametric equivalents were used for continuous variables. Variables that showed imbalance between exposure groups and were associated with asthma were flagged as potential confounders and examined for multicollinearity. Finally, a multiple logistic regression model including these confounders was used to estimate adjusted odds ratios (ORs) with 95% confidence intervals (CIs) for the relationship between maternal age at delivery and the development of asthma in the child. All statistical analyses were conducted by a biostatistician. Stratification variables and survey weights provided by the NSCH were incorporated to account for the complex survey design and ensure national representativeness.

Ethical considerations

This study used publicly available, de-identified data from the NSCH; therefore, institutional review board (IRB) approval was not required.

## Results

**Figure 1:**
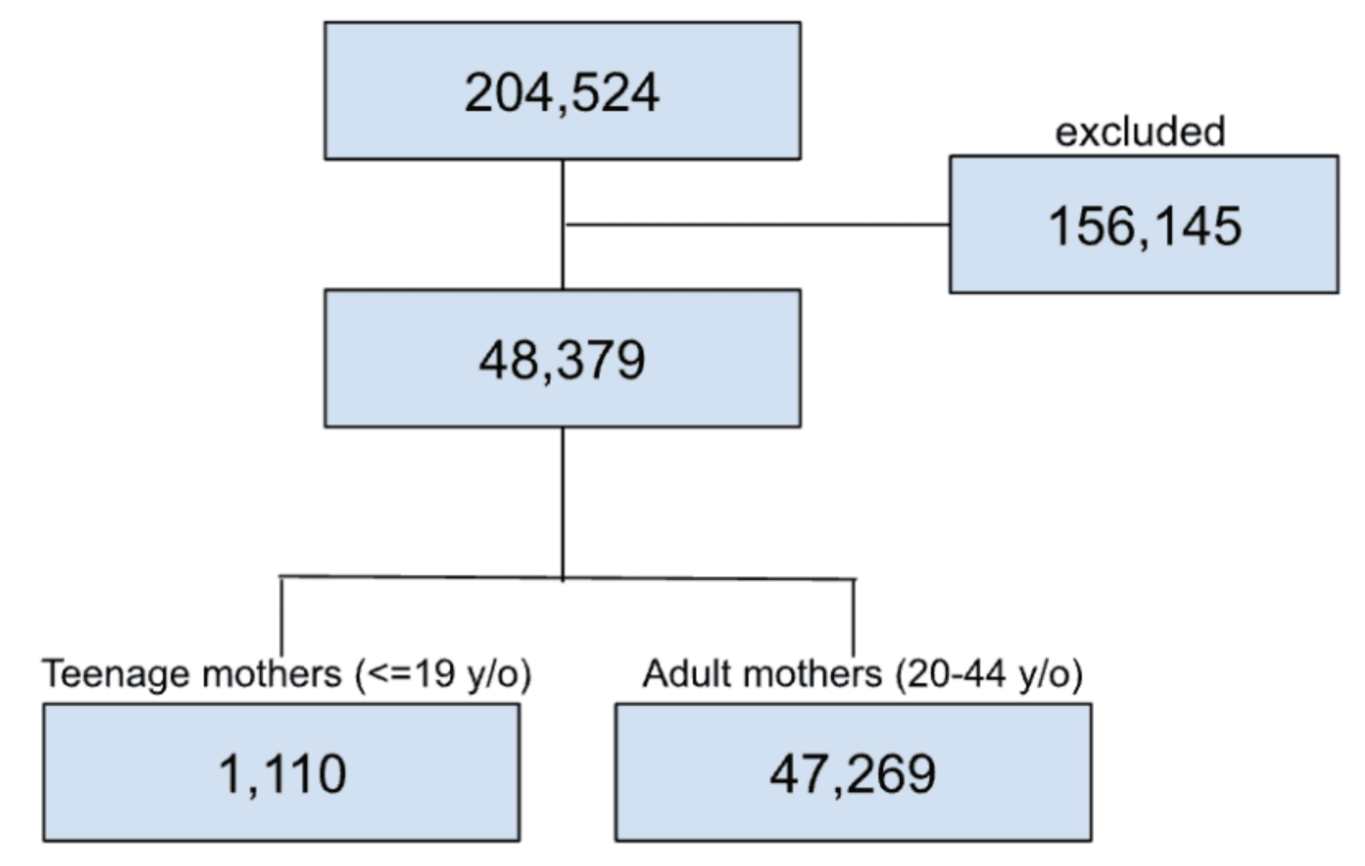
Sample of the study

The total number of eligible participants, based on the demographics of children and mothers, was 204,524. After excluding 156,145 children due to any of the exclusion criteria (children born with congenital birth defects, children with caregivers other than biological/adoptive parents, and missing information in the exposure or the outcome), the final study sample consisted of a total of 48,379 participants. Of the total participants, 1,110 (2.29%) had teenage mothers and 47,269 (97.71%) had adult mothers (see Figure 1). In total, 2,058 participants (4.36%) reported having asthma.

Table 1 shows the distribution of baseline characteristics between teenage mothers and adult mothers, demonstrating differences across different socioeconomic, health, and demographic variables. Children from teenage mothers were more likely to live in single-parent households and have caregivers with lower educational and financial levels as compared to adult mothers. They were also most likely to be exposed to alcohol and drug problems in their households. Additionally, teenage mothers were less likely to breastfeed for extended periods. Lastly, children born to teenage mothers were more likely to have low birth weight and develop epilepsy, depression, anxiety, and blood disorders. This shows that children born to teenage mothers face more health and socioeconomic challenges that may influence health outcomes, as compared to adult mothers. 

**Table 1:** Baseline characteristics of the sample according to the age of the mother at delivery Note: Chi-square test of independence was used to calculate the p-values. N (%) = number and percentage of participants within each group. p-values < 0.05 were considered statistically significant.

Characteristics	Age of the Mother at Delivery		Chi-square (χ²)	p-value
	Adult (20-44) (n = 47,269)	Teen (≤19) (n = 1,110)		
	N (%)	N (%)		
Relation of Caregiver with Child			2,239.42	<0.001
Mother	29,617 (64.14%)	509 (47.88%)	-	-
Father	13,551 (29.35%)	82 (7.71%)	-	-
Other	3,004 (6.51%)	472 (44.40%)	-	-
Marital Status			888.40	<0.001
With Partner	41,161 (89.61%)	641 (60.53%)	-	-
Without Partner	4,771 (10.39%)	418 (39.47%)	-	-
Education Level of Caregiver			1,439.37	<0.001
Less Than High School	1,250 (2.64%)	124 (11.17%)	-	-
High School	4,746 (10.04%)	405 (36.49%)	-	-
Higher Education	12,354 (26.14%)	420 (37.84%)	-	-
College or Graduate Degree	28,919 (61.18%)	161 (14.50%)	-	-
Unable to Provide Basic Needs			314.79	<0.001
Never	31,102 (67.12%)	469 (43.59%)	-	-
Rarely	11,015 (23.77%)	376 (34.94%)	-	-
Somewhat Often	3,417 (7.37%)	192 (17.84%)	-	-
Very Often	802 (1.73%)	39 (3.62%)	-	-
Insurance Coverage (Last 12 Months)			86.36	<0.001
Yes, 12 Months	44,703 (94.85%)	980 (88.61%)	-	-
Yes, but With a Gap	1,116 (2.37%)	52 (4.70%)	-	-
No Health Coverage	1,310 (2.78%)	74 (6.69%)	-	-
Child Born in the US			7.75	0.004
Yes	46,339 (98.60%)	1,074 (97.55%)	-	-
No	658 (1.40%)	27 (2.45% )	-	-
Child Sex			2.87	0.085
Male	24,484 (51.80%)	604 (54.41%)	-	-
Female	22,785 (48.20%)	506 (45.59%)	-	-
Race of Children			172.86	<0.001
White	36,576 (77.38%)	730 (65.77%)	-	-
Black	2,845 (6.02%)	170 (15.32%)	-	-
Other/Multi-racial	7,848 (16.60%)	210 (18.92%)	-	-
Born Preterm?			2.62	0.095
Yes	4,923 (10.49%)	132 (12.05%)	-	-
No	42,012 (89.51%)	963 (87.95%)	-	-
Birth Weight			28.76	<0.001
Born With Very Low Birth Weight	498 (1.08%)	23 (2.18%)	-	-
Born With Low Birth Weight	3,212 (6.99%)	108 (10.24%)	-	-
Normal Birth Weight	42,266 (91.93%)	924 (87.58%)	-	-
Breastfeeding			761.47	<0.001
Never Breastfed	6,788 (14.59%)	468 (42.90%)	-	-
Less Than Six Months	20,370 (43.78%)	472 (43.26%)	-	-
Six Months or Longer	19,370 (41.63%)	151 (13.84%)	-	-
Cigarettes in Household			317.17	<0.001
Yes	5,106 (11.02%)	307 (28.53%)	-	-
No	41,232 (88.98%)	769 (71.47%)	-	-
Overweight Child			1.83	0.141
Yes	793 (1.68%)	25 (2.26%)	-	-
No	46,385 (98.32%)	1,082 (97.74%)	-	-
Diabetes			2.77	0.028
Yes	37 (0.08%)	3 (0.27%)	-	-
No	47,099 (99.92%)	1,107 (99.73%)	-	-
Epilepsy			13.68	<0.001
Yes	284 (0.60%)	17 (1.53%)	-	-
No	46,888 (99.40%)	1,092 (98.47%)	-	-
Frequent Headaches			10.34	<0.001
Yes	122 (0.26%)	9 (0.81%)	-	-
No	47,067 (99.74%)	1,097 (99.19%)	-	-
Tourette Syndrome			0.10	0.226
Yes	13 (0.03%)	1 (0.09%)	-	-
No	47,137 (99.97%)	1,107 (99.91%)	-	-
Depression			74.56	<0.001
Yes	58 (0.12%)	13 (1.18%)	-	-
No	47,104 (99.88%)	1,092 (98.82%)	-	-
Anxiety			42.48	<0.001
Yes	726 (1.54%)	45 (4.07%)	-	-
No	46,439 (98.46%)	1,060 (95.93%)	-	-
Blood Disorders			52.27	<0.001
Yes	273 (0.58%)	26 (2.34%)	-	-
No	46,886 (99.42%)	1,080 (97.66%)	-	-
Alcohol or Drugs Problem in Household			103.24	<0.001
Yes	1,870 (4.09%)	110 (10.54%)	-	-
No	43,895 (95.91%)	934 (89.46%)	-	-

Among the 1,110 children of teenage mothers, 100 (9.01%) had asthma, while among 47,269 children of adult mothers, 2,023 (4.28%) had asthma (p < 0.001). Table 2 demonstrates that the frequency of asthma was significantly higher among children with caregivers who lack partners and have lower socioeconomic status, lower level of education, and more cigarette use. Asthma prevalence was higher in children who reported allergies, obesity, diabetes, epilepsy, headache, depression, anxiety, and blood disorders. Additionally, children who live with individuals who engage in alcohol or drug use have a higher prevalence of asthma. The results also showed that children with asthma have higher rates of preterm birth and low or very low birth weight. Lastly, children who developed asthma were more likely to have never been breastfed or been breastfed for less than six months.

**Table 2:** Baseline characteristics of the sample according to presence or absence of asthma Note: Chi-square test of independence was used to calculate the p-values. N (%) = number and percentage of children within each category. p-values < 0.05 were considered statistically significant.

Characteristics	Asthma in the Child		Chi-square (χ²)	p-value
	No Asthma (n = 46,256)	Asthma (n = 2,123)		
	N (%)	N (%)		
Mother's Age at Delivery			56.69	<0.001
20 to 44 years old	45,246 (95.72%)	2,023 (4.28%)	-	-
≤19 years old	1,010 (90.99%)	100 (9.01%)	-	-
Relation of Caregiver			111.05	<0.001
Mother	28,736 (95.39%)	1,390 (4.61%)	-	-
Father	13,207 (96.88%)	426 (3.12%)	-	-
Other	3,234 (93.04%)	242 (6.96%)	-	-
Marital Status			205.76	<0.001
With Partner	40,183 (96.13%)	1,619 (3.87%)	-	-
Without Partner	4,764 (91.81%)	425 8.19%)	-	-
Education Level of Caregiver			72.36	<0.001
Less than High School	1,285 (93.52%)	89 (6.48%)	-	-
High School	4,869 (94.53%)	282 (5.47%)	-	-
Higher Education	12,118 (94.86%)	656 (5.14%)	-	-
College or Graduate Degree	27,984 (96.23%)	1,096 (3.77%)	-	-
Unable to Provide Basic Needs			198.75	<0.001
Never	30,458 (96.47%)	1,113 (3.53%)	-	-
Rarely	10,772 (94.57%)	619 (5.43%)	-	-
Somewhat Often	3,347 (92.74%)	262 (7.26%)	-	-
Very Often	766 (91.08%)	75 (8.92%)	-	-
Insurance Coverage			1.49	0.474
Yes, 12 Months	43,671 (95.60%)	2,012 (4.40%)	-	-
Yes, but With a Gap	1,114 (95.38%)	54 (4.62%)	-	-
No Health Coverage	1,332 (96.24%)	52 (3.76%)	-	-
Child Born in the US			0.22	0.568
Yes	45,331 (95.61%)	2,082 (4.39%)	-	-
No	658 (96.06%)	27 (3.94%)	-	-
Child Sex			95.13	<0.001
Male	23,767 (94.73%)	1,321 (5.27%)	-	-
Female	22,489 (96.56%)	802 (3.44%)	-	-
Race of Children			163.00	<0.001
White	35,827 (96.04%)	1,479 (3.96%)	-	-
Black	2,747 (91.11%)	268 (8.89%)	-	-
Other/Multi-racial	7,682 (95.33%)	376 (4.67%)	-	-
Born Preterm?			131.16	<0.001
Yes	4,675 (92.48%)	380 (7.52%	-	-
No	41,248 (95.98%)	1,727 (4.02%	-	-
Birth Weight			166.33	<0.001
Very Low Birth Weight	446 (85.60%)	75 (14.40%)	-	-
Low Birth Weight	3,108 (93.61%)	212 (6.39%)	-	-
Normal Birth Weight	41,426 (95.92%)	1,764 (4.08%)	-	-
Breastfeeding			118.51	<0.001
Never Breastfed	6,796 (93.66%)	460 (6.34%)	-	-
Less Than Six Months	19,861 (95.29%)	981 (4.71%)	-	-
Six Months or Longer	18,863 (96.63%)	658 (3.37%)	-	-
Cigarettes in Household			17.93	<0.001
Yes	5,116 (94.51%)	297 (5.49%)	-	-
No	40,226 (95.77%)	1,775 (4.23%)	-	-
Overweight Child			112.35	<0.001
Yes	720 (88.02%)	98 (11.98%)	-	-
No	45,445 (95.74%)	2,022 (4.26%)	-	-
Diabetes			8.37	<0.001
Yes	34 (85.00%)	6 (15.00%)	-	-
No	46,096 (95.62%)	2,110 (4.38%)	-	-
Epilepsy			43.38	<0.001
Yes	264 (87.71%)	37 (12.29%)	-	-
No	45,902 (95.67%)	2,078 (4.33%)	-	-
Frequent Headaches			86.27	<0.001
Yes	103 (78.63%)	28 (21.37%)	-	-
No	46,072 (95.66%)	2,092 (4.34%)	-	-
Tourette Syndrome			14.20	<0.001
Yes	10 (71.43%)	4 (28.57%)	-	-
No	46,133 (95.62%)	2,111 (4.38%)	-	-
Depression			69.70	<0.001
Yes	53 (74.65%)	18 (25.35%)	-	-
No	46,099 (95.65%)	2,097 (4.35%)	-	-
Anxiety			170.66	<0.001
Yes	663 (85.99%)	108 (14.01%)	-	-
No	45,490 (95.77%)	2,009 (4.23%)	-	-
Blood Disorders			10.23	<0.001
Yes	188 (90.82%)	19 (9.18%)	-	-
No	45,998 (95.63%)	2,102 (4.37%)	-	-
Alcohol or Drugs Problem in Household			43.11	<0.001
Yes	1,835 (92.68%)	145 (7.32%)	-	-
No	42,937 (95.78%)	1,892 (4.22%)	-	-

Prior to adjustment, the odds for having asthma were 121.4% higher in children of teenage mothers as compared to those of adult mothers: OR 2.21 (95% CI 1.79-2.73). After adjusting for potential confounders (listed in Table 3), the point estimate decreased but remained significantly higher than 1: OR 1.32 (95%CI 1.03-1.69). In the adjusted model, other variables found associated with increased frequency of asthma were children of teenage mothers, children of mothers without partners, male sex, Black race or multiracial, preterm birth, low and very low birth weight, overweight status, epilepsy, frequent headaches, anxiety, blood disorders, alcohol or drugs problems in household, and never or short duration of breastfeeding.

**Table 3:** Unadjusted and adjusted associations between baseline characteristics and age of a mother and the presence or absence of asthma

Variable	Unadjusted OR (95% CI)	Adjusted OR (95% CI)
Mother's Age at Delivery		
20 to 44 years old	Ref	-
≤19 years old	2.21 (1.79-2.73)	1.32 (1.03-1.69)
Relation of Caregiver		
Mother	Ref	-
Father	0.66 (0.59-0.74)	0.72 (0.64-0.81)
Other	1.54 (1.34-1.78)	1.01 (0.86-1.19)
Marital Status		
With Partner	Ref	-
Without Partner	2.21 (1.98-2.47)	1.59 (1.39-1.81)
Education Level of Caregiver		
Less Than High School	1.76 (1.41-2.21)	1.23 (0.95-1.59)
High School	1.478(1.29-1.69)	1.11 (0.96-1.29)
Higher Education	1.38 (1.25-1.52)	1.13 (1.01-1.26)
College or Graduate Degree	Ref	-
Unable to Provide Basic Needs		
Never	Ref	-
Rarely	1.57 (1.42-1.73)	-
Somewhat Often	2.14 (1.86-2.46)	-
Very Often	2.67 (2.09-3.43)	-
Insurance Coverage		
Yes, 12 Months	Ref	-
Yes, but With a Gap	1.05 (0.79-1.38)	-
No Health Coverage	0.84 (0.64- 1.12)	-
Child Born in the USA		
Yes	Ref	-
No	0.89 (0.60-1.31)	-
Child Sex		
Male	1.55 (1.42-1.70)	1.59 (1.45-1.75)
Female	Ref	-
Race of Children		
White	Ref	
Black	2.36 (2.06-2.70)	1.69 (1.44-1.99)
Other/Multi-racial	1.18 (1.05-1.33)	1.17 (1.03-1.32)
Born Preterm?		
Yes	1.94 (1.73-2.17)	-
No	Ref	-
Birth Weight		
Very Low Birth Weight	3.94 (3.07-5.06)	3.73 (2.86-4.86)
Low Birth Weight	1.60 (1.38-1.85)	1.49 (1.28-1.74)
Normal Birth Weight	Ref	-
Cigarettes in Household		
Yes	1.31 (1.15-1.49)	1.03 (0.89-1.18)
No	Ref	-
Overweight Child		
Yes	3.05 (2.46-3.79)	2.36 (1.86-2.99)
No	Ref	-
Diabetes		
Yes	3.85 (1.61-9.19)	1.59 (0.52-4.83)
No	Ref	-
Epilepsy		
Yes	3.09 (2.18-4.37)	2.02 (1.37-2.97)
No	Ref	-
Frequent Headaches		
Yes	5.98 (3.93-9.11)	3.44 (2.12-5.58)
No	Ref	-
Tourette Syndrome		
Yes	8.74 (2.73-27.89)	1.28 (0.17-9.47)
No	Ref	-
Depression		
Yes	7.46 (4.36-12.76)	1.23 (0.62-2.41)
No	Ref	-
Anxiety		
Yes	3.68 (2.99-4.54)	2.54 (2.02-3.21)
No	Ref	-
Blood Disorders		
Yes	2.21 (1.37-3.55)	1.77 (1.08-2.90)
No	Ref	-
Alcohol or Drugs Problem in Household		
Yes	1.79 (1.50-2.13)	1.27 (1.05-1.55)
No	Ref	-
Breastfeeding		
Never Breastfed	1.49 (1.29-1.72)	1.51 (1.31-1.73)
Less Than Six Months	1.30 (1.16-1.44)	1.30 (1.17-1.45)
Six Months or Longer	Ref	-

Table 1 shows that teenage mothers were significantly more likely to lack a partner, have lower levels of education, and report more socioeconomic challenges compared to adult mothers (p < 0.001 for all comparisons).

As shown in Table 2, asthma was more prevalent in children born to teenage mothers, children with caregivers lacking partners, and those from lower socioeconomic backgrounds.

## Discussion

Our study shows that maternal age at time of birth is associated with the presence of asthma in zero-to-five-year-old children, with an increased prevalence in children born to mothers aged 19 years or less. While current literature primarily focuses on risk factors such as smoking in the household, environmental exposures, and nutritional deficiencies [15], only one study directly isolates maternal age as an independent factor in pediatric asthma [1], while others assess related contributors like maternal stress and socioeconomic status [16,17].

Our study builds on this limited body of evidence by directly examining maternal age as a standalone risk factor, helping fill an important gap in understanding asthma development among children of young mothers. According to the research results, children born to teenage mothers were found to have higher odds of developing asthma, compared to those born to adult mothers. After adjustment for potential confounders, the association remained statistically significant. This reduction in odds - from 2.21 to 1.32 - suggests that part of the relationship is mediated through confounders such as caregiver education, socioeconomic disadvantage, smoking in the home, preterm birth, and low birth weight. However, the association’s persistence after adjustment points to other underlying contributors not fully captured in our model.

This suggests that young maternal age is associated with poorer health outcomes in the child, by mediators different from the potential confounders we were able to adjust for, most likely economic disadvantages, limited access to healthcare, and other adverse social determinants of health that are more prevalent in the younger mother population. Future studies should consider a sequential modeling approach, adding covariates in conceptual blocks, such as socioeconomic status, perinatal factors, and environmental exposures, to better understand which domains most strongly mediate the association between maternal age and childhood asthma.

Teenage mothers are mostly from low-income and socially disadvantaged backgrounds [18]. Furthermore, asthma is a multifactorial disease, and factors such as age, sex, family history, environment, and behaviors are disease triggers [3].

Our findings align with prior evidence linking adolescent motherhood to negative perinatal outcomes and increased pediatric morbidity. Teenage pregnancies have been linked to negative perinatal outcomes, such as low birth weight, reduced Apgar scores, and increased perinatal mortality [19]. The socioeconomic challenges that often accompany adolescent motherhood, including limited financial resources, reduced access to healthcare, and social instability, may further contribute to poorer respiratory outcomes in their children [19]. Moreover, maternal stress during pregnancy, which is more prevalent among teenage mothers, has been shown to dysregulate cortisol levels, a factor associated with wheezing and asthma in early childhood [20]. Epigenetic modifications triggered by prenatal stress may also disrupt the development of the child’s immune and respiratory systems [20]. Taken together, these biological and social stressors likely interact to increase the child’s vulnerability to asthma and other chronic conditions.

In summary, the findings of this study suggest that children born to teenage mothers have a higher risk of developing asthma. The results demonstrate the impact that maternal age can have on a child’s health, with teenage mothers’ children facing an increased asthma burden. This increased risk may be due to factors that disproportionately affect teenage mothers, such as socioeconomic disadvantages, limited access to healthcare, increased environmental stressors, limited educational background, and a higher likelihood of single-parent households. Furthermore, asthma was found to be significantly associated with various health factors in children. Specifically, children with asthma were found to report a lower quality of health as well as other comorbid conditions.

The individuals considered for this study were those who reported to the NSCH, a survey sent to randomly selected households across all US states, minimizing selection bias. However, the self-reported nature of this survey introduces the possibility of recall bias and impacts the reliability of the data used. Although confounders were accounted for, additional unmeasured confounders may still exist. The limited sample used may impact the generalizability of these findings. At the same time, limited sample size can lead to potential underestimation, as is seen in type II errors. Despite the limitations of this study, the findings provide valuable insight into the influence of certain environmental and inherent conditions on the development of asthma, which has been supported by previous studies and urges further exploration.

Our findings highlight the importance of public health interventions that specifically support teenage mothers. Strategies may include early asthma screening for their children, enhanced access to prenatal and postnatal care, and increased caregiver support through education, housing, and social services. Further research should aim to reduce confounding variables and create optimal conditions to determine a more direct relationship between maternal age and other factors considered in this study and the development of asthma in children. While this study did not explore effect modification, future research should examine whether factors, such as race or socioeconomic status, alter the strength or direction of the association between maternal age and asthma. Additionally, longitudinal research on the suggested interventions should be explored to determine the impact and effectiveness of specifically targeting these factors. By addressing these areas, more effective strategies to prevent and manage asthma can be developed, ultimately leading to improved health outcomes.

## Conclusions

This study found that children born to teenage mothers have an increased risk of developing asthma in early childhood compared to those born to adult mothers. After adjusting for a range of confounding factors, this association remained statistically significant, suggesting an independent relationship between younger maternal age and adverse respiratory outcomes. These findings highlight the long-term health implications of adolescent pregnancy and the importance of targeted public health efforts. Interventions such as prenatal education tailored to teenage mothers, early asthma screening in their children, and enhanced postpartum support programs may help mitigate this risk. Supporting teenage mothers through improved access to healthcare, education, and social services can promote better health outcomes for both mothers and their children.
